# Increased susceptibility to cardiovascular effects of dihydrocapcaicin in resuscitated rats. Cardiovascular effects of dihydrocapsaicin

**DOI:** 10.1186/1471-2261-10-39

**Published:** 2010-08-31

**Authors:** Keld Fosgerau, Giuseppe Ristagno, Magdalena Jayatissa, Mads Axelsen, Jacob W Gotfredsen, Uno J Weber, Lars Køber, Christian Torp-Pedersen, Charlotte Videbaek

**Affiliations:** 1 Neurokey AS, Diplomvej 372, DK-2800 Lyngby, Denmark; 2 Mario Negri Institute for Pharmacological Researches, Via La Masa 19, Milan, Italy

## Abstract

**Background:**

Survivors of a cardiac arrest often have persistent cardiovascular derangements following cardiopulmonary resuscitation including decreased cardiac output, arrhythmias and morphological myocardial damage. These cardiovascular derangements may lead to an increased susceptibility towards the external and internal environment of the cardiovascular system as compared to the healthy situation.

**Methods:**

Here we tested the hypothesis that the cardiovascular system in healthy rats and rats resuscitated from a cardiac arrest may be differentially affected by a transient receptor potential vanilloid type 1 agonist, by continuous intravenous infusion of dihydrocapsaicin (DHC).

**Results:**

Compared to baseline, infusion of DHC caused an initial increase in mean arterial blood pressure in both healthy and resuscitated rats of 25% and 10%, respectively. Also, we observed an initial response of tachycardia in both healthy and resuscitated rats of 30% and 20%, respectively. Then, at high levels of DHC infusion (> 2.0 mg/kg/hr) we observed two single episodes of transient bradycardia and hypotension in 33% of the healthy rats, which was consistent with a TRPV1 agonist induced Bezold-Jarisch reflex. In contrast, in resuscitated rats we observed multiple episodes of bradycardia/hypotension in 100% of the rats and at a dose of DHC of 0.65 mg/kg/hr. Notably, this DHC effect could be completely blocked in the resuscitated rats by pre-treatment with atropine, a muscarinic acetylcholine antagonist.

**Conclusions:**

Our results indicate that the susceptibility of the rats towards TRPV1 agonist induced Bezold-Jarisch reflex is increased in those resuscitated from cardiac arrest compared to the healthy situation.

## Background

The transient receptor potential vanilloid type 1 (TRPV1) is a member of the mammalian transient receptor potential family of ion channels [1] and was cloned in 1997 [2]. TRPV1 is a non-selective cation channel with a preference for calcium, which can be directly activated by noxious heat (> 43°C), extracellular acidification, as well as a large heterogeneous group of natural compounds such as dihydrocapsaicin (DHC) from chili pepper [3,4]. The TRPV1 receptor is widely expressed in the human body [5,6] particularly in "port-of-entry" tissues, the central nervous system (CNS), and in the peripheral nervous system in primary small to medium diameter sensory neurons such as dorsal root, trigeminal, and nodose ganglia that give rise to C-fibers and Aδ-fibers [2,7]. The TRPV1 receptor is involved in several biological systems including thermosensation and regulation [8,9] and the sensation of pain [10-12]. Also, in the lung the TRPV1 receptors play an important role in the regulation of respiratory functions [13]. Thus, via stimulation of lung TRPV1 receptors by inhaled irritants may elucidate airway reflexes including cough and bronchoconstriction [13].

We have demonstrated the feasibility of using TRPV1 agonist for obtaining drug-induced mild therapeutic hypothermia in healthy animals (accompanying manuscript). Accordingly this pharmacological approach for obtaining therapeutic hypothermia may prove beneficial in patients resuscitated from a cardiac arrest. On the other hand, a transient receptor potential vanilloid type 1 agonist may differentially affect the cardiovascular system in health and in the compromised situation as following cardiac arrest and cardiopulmunary resuscitation. The heart is richly innervated by sensory and vagal nerve endings. These nerves transduce chemical and mechanical changes from the heart to the brain. Interestingly, sensory nerve endings supplying the heart express TRPV1 [14]. However, the role of TRPV1 receptors in the cardiovascular system is currently not understood and the reported data are equivocal. Thus, it has been shown that administration of capsaicin caused bradycardia and hypotension in anaesthetized dogs or rabbits [15,16], whereas in studies in anaesthetized dogs using intravenous infusion of a chemically pure capsaicin a transient increase in heart rate and blood pressure was demonstrated [17]. Moreover, in anaesthetized guinea pigs it has been observed that capsaicin evokes a biphasic change of heart rate with a prominent bradycardia as an initial component [18] or a triphasic blood pressure response in anesthetized rat following intravenous administration of the TRPV1 agonist anandamide [19]. Interestingly, in TRPV1 receptor knock-out mice injection of capsaicin caused no changes in arterial blood pressure in contrast to the clear response in control mice expressing TRPV1 [20].

Activation of the peripheral termini of TRPV1 receptors causes the release of various pro-inflammatory neuropeptides such as substance P (SP), neurokinin A (NKA), and calcitonin gene-related peptide (CGRP) [4,21], which in turn affect the cardiovascular system. The primary pharmacological action of SP in the isolated spontaneously beating heart is to decrease heart rate and relax coronary resistance vessels [22-24]. The effects of SP on the cardiac system can be blocked by atropine, a competitive antagonist of the muscarinic acetylcholine receptor, showing that it is mediated by the cholinergic nerves [23,25]. On the other hand, the effect of NKA, which is co-stored with SP in capsaicin-sensitive nerves also causes bradycardia, but via both cholinergic and non-cholinergic nerves [18]. In contrast to SP and NKA, GCRP causes tachycardia in isolated guinea pig heart preparations via stimulation of the GCRP receptor [26,27].

In the present study, our goals were to investigate the effect of continuous intravenous infusion of the TRPV1 agonist DHC on the cardiovascular system in conscious rats, and in a model of a compromised cardiovascular system, i.e. the anesthetized rat following cardiac arrest and cardiopulmonary resuscitation. The TRPV1 agonist DHC and infusion paradigm was selected based on feasibility studies of the ability to induce mild therapeutic hypothermia (accompanying manuscript).

## Methods

### Healthy rats

#### Animals

Cardiovascular studies in healthy rats were performed at Covance Inc. (Greenfield, IN, USA) and initiated in 6 male Sprague-Dawley CD/IGS rats (Charles River, Portage, Michigan, USA). Rats were housed under controlled temperature (22 ± 5°C) and humidity conditions (20 - 80%) at a 12 hour normal dark-light cycle (lights on at 06.00 AM) with free access to water and chow (Rodent 2014, Harlan Teklad Global Diets, Indianapolis, IN, USA). The procedures in this study were designed to avoid or minimize discomfort, distress, and pain to animals and in compliance with the U.S. Department of Agriculture's (USDA) Animal Welfare Act (9 CFR Parts 1, 2, and 3), the Guide for the Care and Use of Laboratory Animals (Institute of Laboratory Animal Resources, National Academy Press, Washington, D.C., 1996), and the National Institutes of Health, Office of Laboratory Animal Welfare.

#### Experimental

At least five days prior to infusion the animals were implanted with a DSI Physiotel^® ^Multiplus C50-PXT transmitter (Data Sciences International, St Paul, MN, USA) and a 6-inch beaded polyurethane intravenous catheter (Strategic Applications Inc., Libertyville, IL, USA) set in the jugular vein using aseptic surgical technique. The animals received buprenorphine hydrochloride (0.1 mg/kg, Reckitt Benckiser Pharmaceuticals Inc., Richmond, VA, USA) prior to surgery and ketoprofen (3 mg/kg, Fort Dodge Animal Health, Fort Dodge, IA, USA) following surgery, and were anesthetized with isoflurane (Abbott Laboratories, Abbott Park, IL, USA) throughout the surgical procedure. The DSI system was connected to a data acquisition and analysis system (PONEMAH, Data Sciences International, St. Paul, MN, USA) and enabled to measure systemic arterial pressure and blood temperature. The arterial pressure signal was used to derive systolic, diastolic, and mean arterial pressure as well as heart and respiratory rate. Although data was continuously acquired, derived parameters were collapsed into mean values computed over repetitive logging periods. Data was recorded from 2 hours before starting the infusion until approximately 6.00 AM the following day. A freshly prepared solution of dihydrocapsaicin (0.8 mg/ml, Lot 63908, Clauson Kaas, Farum, Denmark) dissolved in 2% tween 80 (Spectrum Chemicals and Laboratory Products, Gardena, CA, USA) and saline and vehicle control was prepared and sterile filtered before on-set of the infusion. Following a 2 hour settling period and using a KD scientific pump (KD Scientific Inc, Holliston, MA, USA), vehicle control was infused for 1 hour at a flow rate of 3 ml/kg/hr. Next, DHC was infused in 5 steps each lasting 1 hour at the doses of 1.0, 2.0, 2.2, 2.4, and 3.0 mg/kg/hr corresponding to a flow rate of 1.25, 2.5, 2.75, 3.0, and 3.75 ml/kg/hr. Following the termination of the infusion animals were euthanized by CO_2 _inhalation followed by cervical dislocation.

### Cardiac arrested and resuscitated rats

#### Animals

Studies in resuscitated rats were performed at the Weil Institute of Critical Care (Rancho Mirage, CA, USA) in male Sprague-Dawley rats (Harlan Laboratories Inc., station #237, San Diego, CA, USA) aged 6-8 months and weighing 496-523 grams. Animals were housed in groups of 2-3 per cage in a temperature (23 ± 3°C) and humidity (30 - 40%) controlled room and a 12 hour light-dark cycle (lights on at 06.00 AM). Rat diet (NEWCO Distributors, Inc, Rancho Cucamonga, CA, USA) and water were available *ad libitum *except that food was deprived 12 hours prior to surgery. All animals received humane care in compliance with the *Guide for the Care and Use of Laboratory Animals *prepared by the Institute of Laboratory Animal Resources and published by the National Institutes of Health (National Institutes of Health publication 0-309-05337-3, Revised 1996). The protocol was approved by the Institutional Animal Care and Use Committee of the Weil Institute of Critical Care Medicine. The Weil Institute of Critical Care Medicine Laboratories is fully accredited by the Association for Assessment and Accreditation of Laboratory Animal Care International.

#### Preparation and surgery

The animals were anesthetized by an intraperitoneal injection of pentobarbital (45 mg/kg) and additional doses (10 mg/kg) were administrated as required to maintain anesthesia. Antibiotic treatment with cefazolin (250 mg, Moore Medical Corp., New Britain, CT, USA) was administered by intramuscular injection prior to the start of surgery, and a second dose at the end of the experiment. The trachea was orally intubated with a 14 G cannula mounted on a blunt needle with a 145° angled tip (Abbocath-T, Abbott Hospital, North Chicago, IL, USA) as previously described [28]. During surgery animals were spontaneously breathing air and the temperature of the animal was maintained at 37°C ± 0.2°C with the aid of a heating lamp. Blood temperature was measured with a thermocouple microprobe 10 cm in length and 0.5 mm in diameter (9030-12-D-34, Columbus Instruments, Columbus, OH, USA) that was inserted into the right femoral artery and advanced to the distal ascending aorta. A polyethylene catheter (PE-50; Becton-Dickinson, Franklin Lakes, NJ, USA) was advanced into the descending aorta from the surgically exposed left femoral artery for measurement of arterial blood pressure. Electrocardiography (ECG) was continuously monitored by a conventional lead II and end-tidal CO_2 _was continuously monitored with a side-stream infrared CO_2 _analyzer (End-Til IL 200; Instrument Laboratory, Lexington, MA, USA). A polyethylene catheter (PE-50; Becton-Dickinson, Franklin Lakes, NJ, USA) was advanced from the left femoral vein into the inferior cava vein for subsequent infusion of compound. All catheters were flushed intermittently with heparinized saline (2.5 IU/ml of BSA, Western Medical Supple, Arcadia, CA, USA).

#### Cardiac arrest procedure

The cardiac arrest and resuscitation procedure was performed as previously described [29,30]. Briefly, fifteen minutes prior to inducing ventricular fibrillation (VF), baseline measurements were obtained and mechanical ventilation was initiated with an inspired O_2 _fraction (FiO_2_) of 0.21. Ventricular fibrillation was then induced through a guide wire (model C-PMS-301J, Cook Critical Care, Bloomington, IN, USA) advanced from the right jugular vein into the right ventricle. A progressive increase in 60 Hz current to a maximum of 4 mA was delivered to the right ventricular endocardium and maintained for 3 minutes to prevent spontaneous defibrillation. Mechanical ventilation was stopped at the onset of cardiac arrest. After 6 minutes of untreated VF, cardiopulmonary resuscitation (CPR) including pre-cordial chest compressions and mechanical ventilation with a FiO_2 _of 1.0 was initiated. Chest compressions were performed with the aid of a pneumatically driven mechanical chest compressor at a rate of 200 per minute and synchronized to provide a compression-to-ventilation ratio of 2-to-1 with equal compression-relaxation. The depth of compressions was initially adjusted to maintain a coronary perfusion pressure above 23 mmHg and with end-tidal CO_2 _above 11 mmHg. After 6 minutes of CPR, resuscitation was attempted with up to 3 two-joule counter shocks. Return of spontaneous circulation (ROSC) was defined as the return of supraventricular rhythm with a mean aortic pressure above 50 mmHg for at least five minutes. If ROSC was not achieved, a 30 second interval of CPR was performed prior to attempt of a subsequent sequence of up to 3 shocks. The procedure was repeated for a maximum of 3 cycles. If ROSC was not achieved the animal was terminated and excluded from the study.

#### Infusion

Following ROSC animals were allowed to stabilize for 30 minutes and stratified into 2 groups (n = 4) receiving a 6 hour infusion of DHC with or without pre-treatment with atropine (5 mg/kg, Moore Medical Corp., New Britain, CT, USA). Atropine was administered as a 4 IV injections of a 0.4 mg/ml solution every 30 seconds starting 10 minutes prior to the infusion of DHC. A freshly prepared solution of dihydrocapsaicin (0.4 mg/ml, Cat. 92355, Cayman Chemical Company, AH Diagnostics, Aarhus, Denmark) dissolved in 2% tween 80 (Sigma-Aldrich, St. Louis, MO, USA) and saline was prepared and sterile filtered before on-set of the infusion. Then, at t = 0 hours (corresponding to 30 minutes after ROSC) a continuous infusion of DHC was initiated using a Micro Macro XL pump (Abbott Laboratories, Chicago, IL, USA) with a flow rate of 0.4 ml/kg from t = 0 to 30 minutes, then 0.8 ml/kg from t = 30 to 60 minutes, and finally 1.6 ml/kg from t = 1 to 6 hours corresponding to doses of 0.16, 0.33 and 0.65 mg/kg/hr, respectively. Blood temperature and arterial blood pressure were recorded 15 minutes before and then every 15 minutes during the 6 hour infusion. ECG was recorded throughout the infusion. The ambient temperature during the infusion was similar to the conditions during surgery. Following the termination of the infusion animals were euthanized by intraperitoneal injection of pentobarbital (150 mg/kg).

### Statistical analysis

Data are expressed as mean ± standard error (SE) and compared by a one-way or two-way ANOVA and appropriate post-test, unless otherwise stated. P < 0.05 was considered significant. All statistical calculations were performed using GraphPad Prism version 4.00 (GraphPad Software, Inc., San Diego, CA).

## Results

### Healthy rats

#### Clinical observations

Table 1 presents the clinical observations following treatment with vehicle and DHC. All rats survived to study termination, and no changes in clinical condition were observed during infusion with vehicle or during infusion with DHC at the dose of 1.0 mg/kg/hr. Overt DHC-related clinical observations involved changes in activity at doses of 2.0 mg/kg/hr or greater, and piloerection and red pinnae at doses of 2.2 mg/kg/hr or greater. The changes in activity initially consisted of apparent restlessness in all animals within 10 minutes after the start of the 2.0-mg/kg/hr infusion. Other DHC-related observations consisted of piloerection in one rat and red pinnae in all animals at the highest doses of DHC. Vocalization was observed in one rat. Notably, two rats exhibited motor events characterized by increased activity and circling in the cage. Simultaneously cardiovascular events including bradycardia and hypotension were seen, as described below. For both animals, these changes were followed by a period of recumbence and were associated with apparent stiffness of the tail. Shallow respiration and apparent muscle tremors of the thorax were also noted for one rat while it was recumbent. After a short period of 2-3 minutes the animals returned to normal posture.

**Table 1:** Clinical observations in healthy rats.

Dose (mg/kg)	0	1.0	2.0	2.2	2.4	3.0
Step	1	2	3	4	5	6
Mortality	--	--	--	--	--	--
**Clinical Observations**						
Apparent restlessness	--	--	All	--	--	--
Increased activity	--	--	Rat 2	--	--	Rat 1
Circling in cage	--	--	Rat 2	--	--	Rat 1
Stiff tail	--	--	Rat 2	--	--	Rat 1
Lateral recumbency	--	--	Rat 2	--	--	Rat 1
Shallow respiration	--	--	--	--	--	Rat 1
Muscle tremors, thorax	--	--	--	--	--	Rat 1
Vocalizing	--	--	Rat 5	--	--	--
Piloerection	--	--	--	Rat 2	Rat 2	Rat 2
Red pinnae	--	--	--	All	All	All

The table summarizes the clinical observations during the infusion of vehicle and incremental doses of DHC in conscious healthy rats. If no observations were made this was noted as "--", and if an observation was made in all 6 animals this was noted as "All".

#### Cardiovascular parameters, temperature and respiration

**Figure 1:**
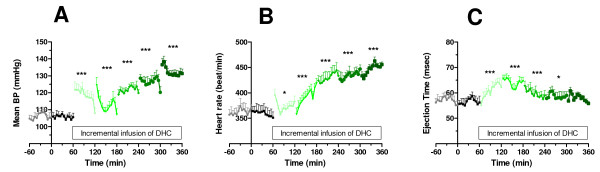
**Mean arterial blood pressure (A) and heart rate (B), and aortic ejection time (C) in healthy conscious rats**. Following baseline characterization animals were infused at t = 0 with vehicle control for 1 hour followed by incremental doses of DHC of 1.0; 2.0; 2.2; 2.4; and 3.0 mg/kg/hr for 1 hour at each condition. Statistics: Each of the doses of DHC were compared to the vehicle control situation by a 1-way ANOVA followed by Dunnett's multiple comparison test ***p > 0.001, **p < 0.01, *p < 0.05. Values are expressed as average ± SE, with n = 6, see Methods for further details.

**Figure 2:**
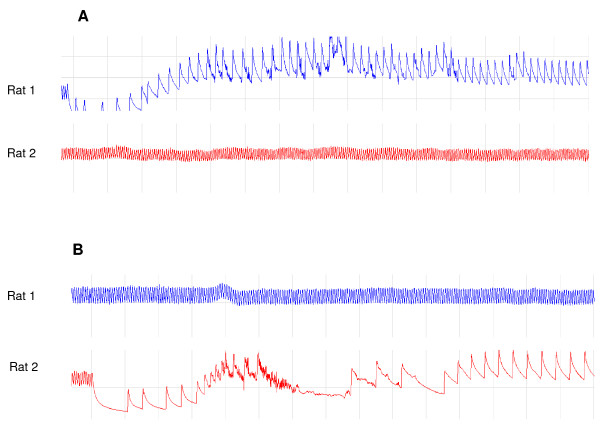
**Curves of mean arterial blood pressure illustrating the episodes of hypotension and bradycardia occurring simultaneously with motor events in Rat 1 (blue) and Rat 2 (red) during the infusion of DHC at (A) t = 360 minutes (i.e. following the end of the 3 mg/kg/hr dose) or (B) at t = 170 minutes (i.e. near the end of the 2 mg/kg/hr dose)**.

**Figure 3:**
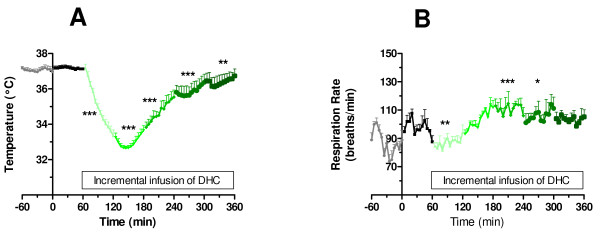
**Blood temperature (A) and respiration rate (B) in healthy conscious rats**. Following baseline characterization animals were infused at t = 0 with vehicle control for 1 hour followed by incremental doses of DHC of 1.0; 2.0; 2.2; 2.4; and 3.0 mg/kg/hr for 1 hour at each condition. Statistics: Each of the doses of DHC were compared to the vehicle control situation by a 1-way ANOVA followed by Dunnett's multiple comparison test ***p > 0.001, **p < 0.01, *p < 0.05. Values are expressed as average ± SE, with n = 6, see Methods for further details.

Generally infusion of DHC caused a dose-dependent increase in mean arterial blood pressure compared to the vehicle control situation (p < 0.001, Figure 1A). Also, we observed a dose-related increase in heart rate, with an increase of approximately 30% compared to vehicle by the end of the last infusion period (p < 0.001, Figure 1B). Aortic ejection time (Figure 1C) was increased compared to vehicle control at all doses of DHC except the final dose of 3.0 mg/kg/hr. However, simultaneously with the motor events described above, we observed cardiac arrhythmias resulting in a marked hypotension and bradycardia (Figure 2). The length of these periods of arrhythmia was approximately 2 minutes, after which the blood pressure and heart rate returned to normal. Hypothermic properties of TRPV1 agonist including DHC have been investigated in separate experiments (accompanying manuscript). Here, body temperature (Figure 3A) displayed a rapid decrease during the first DHC infusion period (1.0 mg/kg/hr) and reached the lowest temperature of 32.7°C, or approximately 4.5°C below baseline, during the infusion of DHC at 2.0-mg/kg/hr. Then, during subsequent infusion periods body temperature increased, but remained below the vehicle control situation until the end of infusion. Derived respiration rate (Figure 3B) were slightly lower during the 1.0 mg/kg/hr infusion and slightly higher during the 2.2 and 2.4 mg/kg/hr infusions.

### Cardiac arrested and resuscitated rats

**Figure 4:**
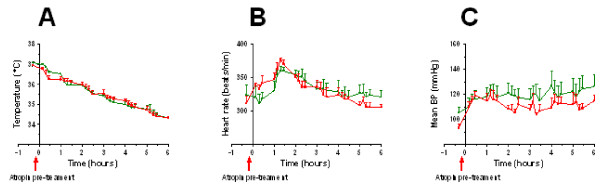
**Blood temperature (A), heart rate (B), and mean arterial blood pressure (C) in rats following a cardiac arrest and cardiopulmonary resuscitation**. Animals were treated with DHC (0.65 mg/kg/hr) and with atropine (5 mg/kg, red line) or without atropine (green line) as pre-treatment. Statistics: We observed no effect of atropine on any of the measured parameters by comparison with a 2-way ANOVA followed by Bonferroni post-test (p = ns). Values are expressed as average ± SE, with n = 4 per group, see Methods for further details.

**Figure 5:**
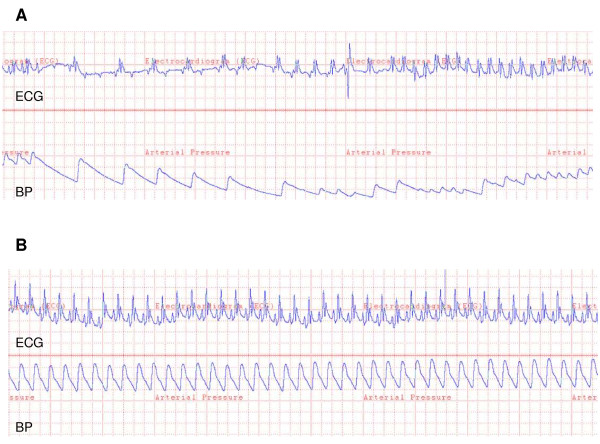
**Representative diagrams of the electrocardiography (ECG) data and arterial blood pressure data in rats following a cardiac arrest, cardiopulmonary resuscitation and return of spontaneous circulation**. (A) Third-degree AV block or complete heart block was as seen in numerous occasions in rats treated with DHC only causing a marked bradycardia and hypotension. (B) Normal ECG as seen in animals pre-treated with atropine.

We observed decrease in mean arterial blood pressure following cardiac arrest and cardiopulmonary resuscitation as compared to the baseline, whereas heart rate and blood temperature was similar (Table 2 and Figure 4C). No differences in baseline conditions before cardiac arrest and also the total time to ROSC and number of shocks when comparing DHC treated animals pre-treated with or without atropine (Table 2, p = ns). Electrocardiography (ECG) and arterial blood pressure tracings in resuscitated rats are shown in Figure 5. In all the rats treated with DHC only, we observed several short-lasting episodes of bradycardia associated with hypotension. The episodes lasted from approximately 10 - 100 seconds and were observed about 5-10 times during the entire course of the infusion of DHC, especially in the first hour. The ECG findings were consistent with a third-degree AV block with no apparent relationship between P waves and QRS complexes (Figure 5A).

**Table 2:** Baseline characteristics in rats resuscitated from cardiac arrest.

	DHC only	DHC + atropine
Bodyweight (g)	504 ± 8	504 ± 8
PaO_2 _(mmHg)	85 ± 4	81 ± 1
PaCO_2 _(mmHg)	39 ± 4	40 ± 4
pH	7.44 ± 0.02	7.45 ± 0.02
Blood temperature (°C)	37.0 ± 0.1	37.0 ± 0.1
Heart rate (beats/min)	333 ± 18	348 ± 8
Mean arterial blood pressure (mmHg)	145 ± 8	140 ± 4
ROSC attempts (n)	2.5 ± 1.2	2.0 ± 0.8
Shocks (n)	7 ± 4	7 ± 4
Total time to ROSC (min)	13.8 ± 1.0	13.3 ± 0.7

The table summarizes the baseline characteristics in rats prior to cardiac arrest and resuscitation. Also the number of shocks and ROSC attempts and total time to ROSC are given prior to the treatment with DHC and with or without atropine. Statistics: We observed differences between the two study groups on any of the measured parameters by comparison with a student's t-test (p = ns). Values are expressed as average ± SE, with n = 4 per group, see Methods for further details.

In animals treated with atropine prior to the infusion of DHC the occurrence of a third-degree AV block episodes and associated bradycardia and hypotension was completely absent during the entire infusion period. Also pre-treatment with atropine did not change the effect of DHC on body temperature, heart rate and mean arterial blood pressure (p = ns, Figure 4). Infusion of DHC caused a decrease in blood temperature from the 37°C at baseline reaching the minimum of 34°C at the end of the 6 hour infusion (Figure 4A). Also, we observed an initial increase in heart rate of approximately 10% within the first 2 hours of infusion (Figure 4B), which then declined towards the end of the infusion back to baseline level. Finally, we observed a minor increase in mean arterial blood pressure of approximately 20% (Figure 4C) during the infusion of DHC compared to the baseline level of approximately 100 mmHg.

## Discussion

On two occasions in the healthy rats we observed a transient cardiovascular episode of bradycardia and hypotension accompanied by a motor component during the infusion of DHC, a TRPV1 agonist. Saito and Yamamoto reported that oral administration of capsaicin to rats caused tremor, clonic convulsion, dyspnea and lateral or prone position before death, and speculated that the cause of death may be associated to hypotension [31]. In anaesthetized artificially ventilated dogs administered intravenously with capsaicin elicited the Bezold-Jarisch reflex [32], which is characterized by hypotension and bradycardia [33]. Moreover, studies in TRPV1 receptor knock-out mice have emphasized the role of the TRPV1 receptors in the activation of the Bezold-Jarisch reflex [20]. Here we report transient episodes of bradycardia and hypotension, which was accompanied by convulsions/spasmodic movements. Also in pilot dose finding experiments during infusion of very high levels of DHC to healthy rats, we observed convulsions or spasmodic movements followed by death in about 20% of the tested rats (data not shown). While we cannot exclude that the origin of the cardiovascular collapse observed might be ascribed to unidentified factors causing hypotension and bradycardia, collectively our observations are consistent with an activation of the Bezold-Jarish reflex by stimulation of the TRPV1 receptor with the agonist DHC.

This is the first study to characterize the cardiovascular profile of a TRPV1 agonist in a model of a compromised cardiac system - the resuscitated rats. The results indicate that the susceptibility of the resuscitated cardiac arrest rats towards TRPV1 agonist induced Bezold-Jarisch reflex is increased compared to the healthy situation. The duration of the episodes of bradycardia/hypotension was relatively short in the resuscitated rats compared to the healthy rats. On the other hand 100% of the resuscitated rats compared to 33% of the healthy rats displayed episodes of bradycardia/hypotension. Also, the episodes occurred at lower levels of DHC in the resuscitated cardiac arrest animals compared to the healthy rats. The reason for this difference is not known, but again may relate to the structural and functional differences of the myocardium in the two models. Cardiac arrest causes a decrease in myocardial contractility [34] and persistent cardiovascular derangements following CPR is seen including decreased cardiac output, arrhythmias and morphological myocardial damage [35]. The ischemia and associated myocardial damage leads to an increase of CO_2 _and lactic acid in the interstitial compartment, which in turn cause cellular acidosis and a decrease in pH [36]. Accordingly, buffer agents administered during CPR may ameliorate post-resuscitation myocardial dysfunction and thereby improve survival [37]. Since the TRPV1 receptor is sensitized by extracellular protons [38], the observed increased susceptibility of the resuscitated rats versus the healthy rats towards DHC-induced episodes of bradycardia and hypotension therefore may be from a difference in the myocardial pH in the two situations. However, we did not measure myocardial pH in our experiment, and therefore we cannot exclude that other factors such as the level of anesthesia or the rate of infusion may have impacted on the reported results.

Intravenous infusion of capsaicin caused a dose-dependent increase in heart rate and blood pressure in anaesthetized dogs [17]. Here, using DHC, we confirm these results in healthy conscious rats and in resuscitated rats and show that arterial blood pressure and heart rate increased by intravenous infusion. Similarly we have observed tachycardia and hypertension in response to intravenously infused DHC in conscious calves (data not shown). This chronotropic effect of DHC may be related to the release of GCRP following activation of the TRPV1 receptor [26,27]. Using the design of a step-wise incremental dose of DHC by increasing the infusion rate we here demonstrate that the general tachycardia/hypertension precedes the episodes of bradycardia/hypotension in the healthy rats. Accordingly the episodes of bradycardia/hypotension, which in turn may be mediated by the release of NKA and SP (20, 27) may occur at higher level of TRPV1 activation: i.e., the biphasic or triphasic change of heart rate previously reported following administration of a TRPV1 agonist [18,19] may be a result of pharmacokinetic properties.

In the healthy rats, the blood pressure changes during the 2.0-mg/kg/hr infusion were not dose proportional. During this infusion period, body temperature reached its lowest level (approximately 4.5°C lower than vehicle treatment). Because central control of blood pressure is influenced by body temperature, the substantial decrease in body temperature observed during the 2.0-mg/kg/hr infusion may be responsible for the lack of dose proportionality noted in the blood pressure changes. Ejection time would normally be shorter in response to higher heart rate and longer during lower heart rates. In this experiment, heart rate was higher, but ejection time also increased. This could be a predictor of a loss of cardiac contractility.

Interestingly, the episodes of bradycardia and hypotension and third degree AV block elucidated by DHC in the resuscitated situation could be fully prevented by pre-treatment with the muscarinic acetylcholine receptor antagonist atropine. It has been previously reported that the cardiovascular effects of SP [23,25], but not of NKA [18], could be prevented by atropine. Our data emphasizes the role of the cholinergic nerves in the mediation of the Bezold-Jarish reflex. It is not known whether the observed cardiac effects were a result of a direct stimulation of the vagus nerve, which do express TRPV1 [14], or occurs indirectly with the vagus nerve transmitting a signal generated via stimulation of TRPV1 receptors in the CNS, and this may be subject for further experimentation.

## Conclusions

In conclusion, our results demonstrate that intravenous infusion of the TRPV1 agonist DHC to healthy rats causes an initial hypertension and tachycardia. At high levels of DHC this may be followed by transient episodes of bradycardia and hypertension, the so-called Bezold-Jarisch reflex. We also demonstrate that the resuscitated cardiac arrest situation has a higher susceptibility towards this reflex, which can be blocked by pre-treatment with the muscarinic acetylcholine receptor antagonist atropine. Clinically our results indicate that while infusion of DHC may induce mild hypothermia relevant for the treatment of cardiac arrest patients this may be accompanied by an increased number of cardiac events and need of relevant pre-treatment.

## Competing interests

The study was sponsored by Neurokey AS. At the time of the study K. Fosgerau, M Jayatissa, M. Axelsen^1^, JW Gotfredsen, UJ Weber and C Videbaek where employed at the company. JW Gotfredsen, UJ Weber, L Køber and C Torp-Pedersen were founders of the company.

## Authors' contributions

All authors have read and approved the final manuscript. KF: have made substantial contributions to conception and design, or acquisition of data, or analysis and interpretation of data, and writing of article. GR, MJ, CV: Have made contributions to conception and design, or acquisition of data in cardiac arrest rats. MA, JG, UW, LK, CT: have been involved in drafting the manuscript or revising it critically for important intellectual content.

## Pre-publication history

The pre-publication history for this paper can be accessed here:

http://www.biomedcentral.com/1471-2261/10/39/prepub
